# Guidelines, Editors, Pharma And The Biological Paradigm Shift

**DOI:** 10.4103/0973-1229.32176

**Published:** 2007

**Authors:** Ajai R. Singh, Shakuntala A. Singh

**Affiliations:** * *Editor, MSM*; ** *Deputy Editor, MSM.*

**Keywords:** *Academia*, *Pharmaceutical Industry*, *Clinical Practice Guidelines*, *Best Practice Guidelines*, *Academic Medical Centers*, *Medical Associations*, *Research Journals*, *Clinical Research*, *Public Welfare*, *Pharma Image*, *Corporate Welfare*, *Biological Psychiatry*, *Law Suits Against Industry*

## Abstract

Private investment in biomedical research has increased over the last few decades. At most places it has been welcomed as the next best thing to technology itself. Much of the intellectual talent from academic institutions is getting absorbed in lucrative positions in industry. Applied research finds willing collaborators in venture capital funded industry, so a symbiotic growth is ensured for both.

There are significant costs involved too. As academia interacts with industry, major areas of conflict of interest especially applicable to biomedical research have arisen. They are related to disputes over patents and royalty, hostile encounters between academia and industry, as also between public and private enterprise, legal tangles, research misconduct of various types, antagonistic press and patient-advocate lobbies and a general atmosphere in which commercial interest get precedence over patient welfare.

Pharma image stinks because of a number of errors of omission and commission. A recent example is suppression of negative findings about Bayer's Trasylol (Aprotinin) and the marketing maneuvers of Eli Lilly's Xigris (rhAPC). Whenever there is a conflict between patient vulnerability and profit motives, pharma often tends to tilt towards the latter. Moreover there are documents that bring to light how companies frequently cross the line between patient welfare and profit seeking behaviour.

A voluntary moratorium over pharma spending to pamper drug prescribers is necessary. A code of conduct adopted recently by OPPI in India to limit pharma company expenses over junkets and trinkets is a welcome step.

Clinical practice guidelines (CPG) are considered important as they guide the diagnostic/therapeutic regimen of a large number of medical professionals and hospitals and provide recommendations on drugs, their dosages and criteria for selection. Along with clinical trials, they are another area of growing influence by the pharmaceutical industry. For example, in a relatively recent survey of 2002, it was found that about 60% of 192 authors of clinical practice guidelines reported they had financial connections with the companies whose drugs were under consideration. There is a strong case for making CPGs based not just on effectivity but cost effectivity. The various ramifications of this need to be spelt out. Work of bodies like the Appraisal of Guidelines Research and Evaluation (AGREE) Collaboration and Guidelines Advisory Committee (GAC) are also worth a close look.

Even the actions of Foundations that work for disease amelioration have come under scrutiny. The process of setting up ‘Best Practices’ Guidelines for interactions between the pharmaceutical industry and clinicians has already begun and can have important consequences for patient care. Similarly, Good Publication Practice (GPP) for pharmaceutical companies have also been set up aimed at improving the behaviour of drug companies while reporting drug trials

The rapidly increasing trend toward influence and control by industry has become a concern for many. It is of such importance that the Association of American Medical Colleges has issued two relatively new documents - one, in 2001, on how to deal with individual conflicts of interest; and the other, in 2002, on how to deal with institutional conflicts of interest in the conduct of clinical research. Academic Medical Centers (AMCs), as also medical education and research institutions at other places, have to adopt means that minimize their conflicts of interest.

Both medical associations and research journal editors are getting concerned with individual and institutional conflicts of interest in the conduct of clinical research and documents are now available which address these issues. The 2001 ICMJE revision calls for full disclosure of the sponsor's role in research, as well as assurance that the investigators are independent of the sponsor, are fully accountable for the design and conduct of the trial, have independent access to all trial data and control all editorial and publication decisions. However the findings of a 2002 study suggest that academic institutions routinely participate in clinical research that does not adhere to ICMJE standards of accountability, access to data and control of publication.

There is an inevitable slant to produce not necessarily useful but marketable products which ensure the profitability of industry and research grants outflow to academia. Industry supports new, not traditional, therapies, irrespective of what is effective. Whatever traditional therapy is supported is most probably because the company concerned has a product with a big stake there, which has remained a ‘gold standard’ or which that player thinks has still some ‘juice’ left.

Industry sponsorship is mainly for potential medications, not for trying to determine whether there may be non-pharmacological interventions that may be equally good, if not better. In the paradigm shift towards biological psychiatry, the role of industry sponsorship is not overt but probably more pervasive than many have realised, or the right thinking may consider good, for the health of the branch in the long run.

An issue of major concern is protection of the interests of research subjects. Patients agree to become research subjects not only for personal medical benefit but, as an extension, to benefit the rest of the patient population and also advance medical research.

We all accept that industry profits have to be made, and investment in research and development by the pharma industry is massive. However, we must also accept there is a fundamental difference between marketing strategies for other entities and those for drugs.

The ultimate barometer is patient welfare and no drug that compromises it can stand the test of time. So, how does it make even commercial sense in the long term to market substandard products? The greatest mistake long-term players in industry may make is try to adopt the shady techniques of the upstart new entrant. Secrecy of marketing/sales tactics, of the process of manufacture, of other strategies and plans of business expansion, of strategies to tackle competition are fine business tactics. But it is critical that secrecy as a tactic not extend to reporting of research findings, especially those contrary to one's product.

Pharma has no option but to make a quality product, do comprehensive adverse reaction profiles, and market it only if it passes both tests.

Why does pharma adopt questionable tactics? The reasons are essentially two:

1. What with all the constraints, a drug comes to the pharmacy after huge investments. There are crippling overheads and infrastructure costs to be recovered. And there are massive profit margins to be maintained. If these were to be dependent only on genuine drug discoveries, that would be taking too great a risk.
2. Industry players have to strike the right balance between profit making and credibility. In profit making, the marketing champions play their role. In credibility ratings, researchers and paid spokes-persons play their role. All is hunky dory till marketing is based on credibility. When there is nothing available to make for credibility, something is projected as one and marketing carried out, in the calculated hope that profits can accrue, since profit making must continue endlessly. That is what makes pharma adopt even questionable means to make profits.

Essentially, there are four types of drugs. First, drugs that work and have minimal side-effects; second, drugs which work but have serious side-effects; third, drugs that do not work and have minimal side-effects; and fourth, drugs which work minimally but have serious side-effects. It is the second and fourth types that create major hassles for industry. Often, industry may try to project the fourth type as the second to escape censure.

The major cat and mouse game being played by conscientious researchers is in exposing the third and fourth for what they are and not allowing industry to palm them off as the first and second type respectively. The other major game is in preventing the second type from being projected as the first. The third type are essentially harmless, so they attract censure all right and some merriment at the antics to market them. But they escape anything more than a light rap on the knuckles, except when they are projected as the first type.

What is necessary for industry captains and long-term players is to realise:

1. Their major propelling force can only be producing the first type. 2. They accept the second type only till they can lay their hands on the first. 3. The third type can be occasionally played around with to shore up profits, but never by projecting them as the first type. 4. The fourth type are the laggards, real threat to credibility and therefore do not deserve any market hype or promotion.

In finding out why most pharma indulges in questionable tactics, we are lead to some interesting solutions to prevent such tactics with the least amount of hassles for all concerned, even as both profits and credibility are kept intact.

